# Automated Benchmarking of Variable‐Property Soft Robotic Fingertips to Enable Task‐Optimized Sensor Selection

**DOI:** 10.1002/advs.202509991

**Published:** 2025-08-20

**Authors:** David Hardman, Benhui Dai, Qinghua Guan, Antonia Georgopoulou, Fumiya Iida, Josie Hughes

**Affiliations:** ^1^ Bio‐Inspired Robotics Lab Department of Engineering University of Cambridge Cambridge CB2 1PZ UK; ^2^ CREATE Lab EPFL Lausanne CH‐1015 Switzerland; ^3^ Soft Materials Laboratory EPFL Lausanne CH‐1015 Switzerland

**Keywords:** lifetime timescales, robotics, sensor materials, sustainability

## Abstract

Tactile fingertips are a vital component of biological dexterity, where they convey information from the environment through our sensory systems. Similarly, sensorized robotic fingertips are needed to unlock robotic dexterity, versatility, and diverse interactions, which remain significant interdisciplinary challenges. This potential means that hundreds of materials, transducers, and geometries are being developed for soft robotic sensing, but there are very few ways by which they can be compared: a lack of characterizations of the rich interplay between different sensor morphologies, form factors, sensing technologies, material softnesses, and viscosities means that the full solution space is rarely explored. In this work, 15 identically‐shaped robotic fingertips are benchmarked by a fully automated system, covering eight different materials and six broadly‐ranging sensing mechanisms. Diverse mechanical and sensory datasets are collected over a 30 min runtime, designed around five task‐optimized characterization axes. Among these, findings include sensitivities to forces below 0.1 N, ninefold increases in response to human touches, and 0.88 mm localization across a single‐material soft 3D fingertip using electrical impedance tomography. Optimizable tasks are demonstrated via self‐configuration of a two‐finger robotic gripper. The self‐configurable pipeline also enables autonomous adaptability: how robotic manipulators can be optimized over task, environmental, and lifetime timescales is discussed.

## Graphical abstract

Sensorized fingertips are needed to unlock robotic dexterity, versatility, and diverse interactions. Here, 15 identically‐shaped robotic fingertips are benchmarked by a fully automated toolchange system, covering eight different materials and six broadly‐ranging sensing mechanisms. Among these, findings include sensitivities to forces below 0.1 N, ninefold increases in response to human touches, and 0.88 mm localization across a single‐material soft fingertip.

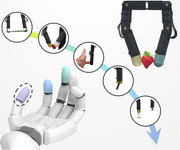

## 1 Introduction

In natural organisms, tactile sensing has evolved to facilitate dexterity across a broad range of tasks and environments.^[^ ^1^, ^2^, ^3^ ^]^ In humans, the perception of sensory data from our fingertips plays a large role in the complexity of our environmental interactions. Any variations in fingertip stiffness directly affect our manipulation and perception capabilities,^[^ ^4^, ^5^ ^]^ which change over our lifetimes.^[^ ^6^, ^7^ ^]^ In primates, the fingertip density of mechanoreceptors varies across species.^[^ ^8^ ^]^ As we look to design sensorized fingertips for increasingly capable robotic hands, humanoids, and grippers, we might therefore expect to change their mechanical and sensor properties with different tasks, environments, and lifetimes. However, there is currently no recognized approach by which the hundreds of available materials, transducers, and morphologies can be tested, compared, and selected. As such, the combination of dexterity and versatility for embodied agents remains an unresolved cross‐disciplinary challenge.^[^ ^9^, ^10^ ^]^

In 1996, Shimoga and Goldenberg manually characterized the mechanical properties of six non‐sensorized robotic fingertips, using their results to argue the necessity of soft materials.^[^ ^11^ ^]^ Since then, numerous sensorized robotic fingertips have been developed, with core technologies including resistive, magnetic, vision‐based, and pneumatic. In the literature, each new fingertip uses a different form‐factor and set of functional materials. As a result, each new sensing type and implementation has a unique set of embodied characteristics, and it is challenging to make an optimized selection without task‐specific physical testing.^[^ ^12^, ^13^, ^14^, ^15^, ^16^, ^17^ ^]^ A sensor's mechanical properties not only affect its force‐deformation profile, but also act as a filter for the information which can physically be received from the environment.^[^ ^18^ ^]^ Each sensory transducer also filters information in this way: pneumatic chambers and magnetic monitoring may filter out the capacitive effect of human touch, while the stability of capacitive sensors could filter out changes in temperature. In multi‐fingered grippers, the number of available material, transducer, and morphology combinations quickly explodes: a typical solution is to use identical tips on every finger, rather than exploiting the custom information filtering which could be made possible by selecting different fingertips for each new task.

**Figure 1:**
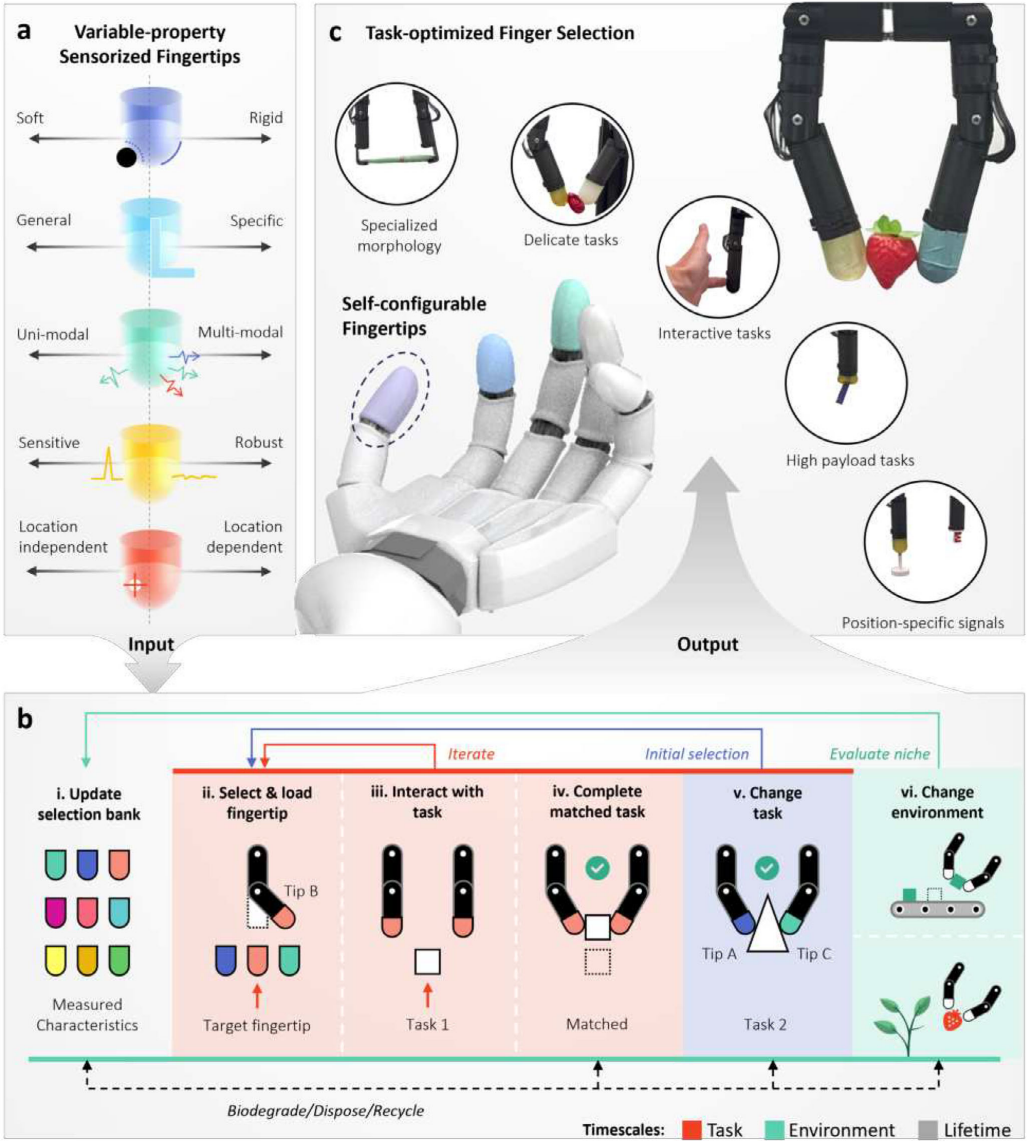
Characterizing self‐configurable sensorized fingertips in a robotic manipulator. a) We automatically benchmark 15 modular fingertips across five selection axes. b) Using the toolchange mechanism, fingertips can be selected and installed without human intervention, optimized to specific tasks and environments. Additionally, damaged fingertips can be renewed, recast and recycled at the end of their lifetime without any changes to their surrounding mechanical and electrical systems. c) We show that multiple sensing modalities and specialized tasks can be accomplished with a simple robotic finger structure, matching the selected fingertips to each particular task using their benchmarked characteristics.

In this work, we propose and develop a standardized platform on which robotic fingertips containing completely different materials and sensing mechanisms can be automatically evaluated: we benchmark the characteristics of 15 separate robotic fingertips which we fabricate to have identical form factors (**Figure** **1**a), containing a mix of sensing technologies and materials. In doing so, we build a dataset for a bank of 15 options that enables fingertips to be individually selected for a specific task, environment, or lifetime: Figure 1b. By implementing an automatic toolchange mechanism, our robotic gripper can self‐configure every fingertip which it uses, providing a route for fully‐automated selective information filtering. With our benchmarking platform, different soft robotic materials and sensing technologies can be directly compared and contrasted: a step which has previously been a bottleneck when selecting components for multimodal manipulation and dexterity. We provide the initial dataset of 15 characterized fingertips in the supplementary material, as well as details of the automated tests, and CAD files such that more fingertips can be fabricated and compared. As the dataset of benchmarked fingertips continues to grow, the adaptability of our automated platform means that new characterization tests (such as shape classification during grasping^[^ ^19^ ^]^ or material recognition^[^ ^20^ ^]^) could be introduced by a wide community, providing a valuable resource for design, fabrication, and task optimization.

Physically benchmarking robotic components for manipulation tasks eliminates the uncertainty of a Sim2Real gap, and ensures that any characterized datasets used for fingertip selection reflect the specific nonlinearities, form factors, fabrication defects, and time dependencies of each fingertip.^[^ ^21^, ^22^ ^]^ This is a particular benefit when characterizing soft sensor morphologies, though existing examples tend to focus on individual sensors in a given run.^[^ ^23^, ^24^ ^]^ The self‐configurability which we introduce instead allows multiple fingertips to be automatically tested and selected in quick succession. Though previous works and industry implementations have explored the automated fabrication and loading of passive robotic fingers,^[^ ^25^ ^]^ this work showcases the first example of fully automated and self‐configurable soft sensors for task‐optimized fingertip benchmarking and selection.

The benefits and technologies behind modular robotic approaches are well‐explored in the literature.^[^ ^26^, ^27^ ^]^ Examples include: reconfigurable hands with single sensing mechanisms^[^ ^28^, ^29^ ^]^; self‐configurability in a modular centipede robot^[^ ^30^ ^]^; and interchangeable fingertip‐sized magnetic sensor skins which can be used to facilitate human‐to‐robot skill transfer.^[^ ^31^ ^]^ Within soft robotics, modularity provides adaptability between different tasks through voxel reconfiguration,^[^ ^32^, ^33^ ^]^ with the optional integration of embedded and embodied sensing modules for perception.^[^ ^34^, ^35^ ^]^

Compared to existing studies on sensorized fingertips, the modularity which we introduce to our device enables entirely different sensing mechanisms to be automatically selected for specific tasks, rather than focusing on only one solution.^[^ ^12^, ^15^, ^17^, ^36^, ^37^ ^]^ As shown in Figure 1b, our proposed set of self‐configurable benchmarked fingertips creates adaptability across three timescales in robotic manipulation: task (in which fingertips can be automatically interchanged to selectively filter information), environment (in which key mechanical and sensor characteristics can be continuously varied within changing surroundings), and lifetime (in which worn‐down fingertips can be replaced and biodegraded without pausing the system's functionality). Coupled with the existence of biodegradable soft sensor materials,^[^ ^38^, ^39^ ^]^ this lifetime timescale provides a pipeline for additional sustainability within the proposed system: fingertips designed for single‐use disposal or with worn‐down contact surfaces could be directly biodegraded without having to make any additional changes to the gripper. To demonstrate the compatibility of our self‐configurable pipeline with soft sustainable sensors, three of the 15 benchmarked fingertips are fabricated from sustainable materials. One biodegradable ionically‐conductive composition is comprised of gelatin, glycerol, water, citric acid, and sodium chloride.^[^ ^40^ ^]^ The remaining two organogel fingertips are based on a deep eutectic solvent (DES) of glycerol and choline chloride, which is considered a green sustainable solvent.^[^ ^41^, ^42^ ^]^ In contrast to other conductive fillers such as intrinsic conductive polymers and metallic fillers, this DES is biodegradable, non‐toxic, and does not introduce hazardous nanoscale particles into the biome after the product's end of life.^[^ ^43^ ^]^ In addition, the network of hydrogen bonds that forms between glycerol and choline chloride stabilize the gel and reinforce its mechanical properties.^[^ ^44^, ^45^ ^]^

Alongside 12 alternatives, we automatically benchmark these three sustainable fingertips across the five task‐optimized selection axes of Figure 1a, so that they can be included for use in Figure 1b's selection bank across changing tasks and environments. Their datasets are built upon the collection of mechanical properties and force sensitivities in three discrete orientations, temperature responsivity, and human interactability. Localization benchmarking is performed for the sustainable materials, using electrical impedance tomography to sensorize their unimpeded 3D surfaces. Using this approach, we demonstrate the sub‐millimeter resolution of a 4 mm diameter insulating probe pressed into the surface of a hydrogel fingertip. Throughout this work, we demonstrate how the variable properties of the sensorized fingertips can be exploited for a range of manipulation tasks in a two‐finger gripper (Figure 1c): fruit picking, guided human/robot interaction, contactless temperature responsivity, localized payload delivery, and force control are performed.

## 2 Setup Overview

To benchmark a complete set of self‐configurable fingertips, we first develop a consistent geometry (detailed fully in Figure S1, Supporting Information): a cylindrical body with a diameter of 25 mm and height of 20 mm, terminated with a hemisphere of 12.5 mm radius. The base of each fingertip contains a custom toolchange mechanism, which allows it to connect to an actuated robotic finger. The finger can automatically unload, install, and communicate with 15 unique fingertips based on the task and environment being faced (Video S1, Supporting Information). All driving motors are contained within the fingertip, such that the design can be scaled to multi‐finger implementations.

**Figure 2:**
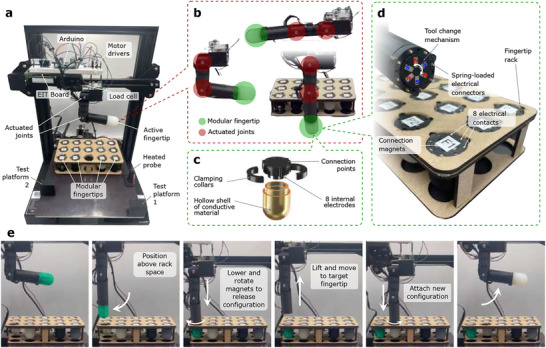
Single‐finger benchmarking platform. a) The actuated finger above a rack of 15 identically‐shaped fingertips, which can all be automatically installed. b) Three configurations with the actuated joints at their mechanical limits. c) An exploded view of a single material fingertip structure, used to benchmark three sustainable materials and three conductive elastomers. d) Close‐up view of the mechanical and electronic connection interface. e) The automated toolchange mechanism for replacing sensorized fingertips, creating both mechanical and electrical connections in 22 s (Video S1, Supporting Information).

**Figure** **2**a,b introduces the modular toolchange setup in a single‐finger configuration, which is used to characterize the rack of 15 different fingertips examined in this work. As shown in Figure 2b, each finger contains three internal N20 motors to actuate its motion: two actuated joints allow it to bend, while one motor controls the automated toolchange mechanism which can freely load and unload any of the fingertips, while using spring‐loaded connectors to join up to eight electrical connections for sensor readings (Figure 2d and Section 5). The controlling electronics (an Arduino, EIT board, and motor drivers) are kept external, which aids scalability to multi‐finger implementations, such as the self‐configurable dual‐finger gripper introduced in Figure 1. Before sensorization is introduced, the configurable nature of this gripper enables task‐specific fingertips to be automatically selected and loaded to match each task: Video S1 (Supporting Information) demonstrates how soft fingertips used for delicate fruit picking can be rapidly exchanged for custom‐morphology ‘scoopers’ which can easily grasp otherwise difficult items such as a horizontal pen.

**Figure 3:**
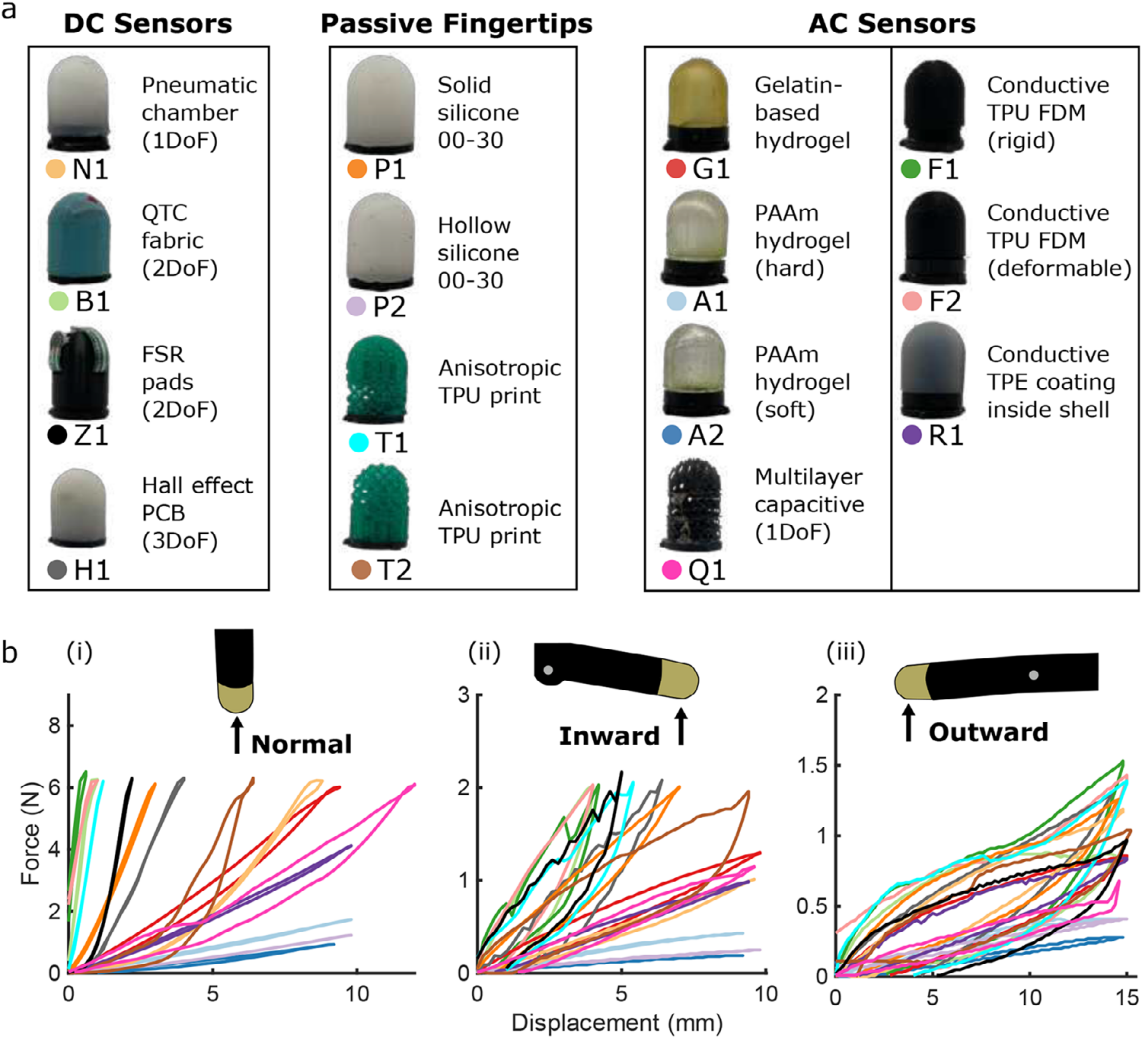
Details of all 15 benchmarked fingertips. a) Brief descriptions of the 15 fingertips: 4 passive, 4 DC sensorized, and 7 AC sensorized. b) The mechanical characteristics of all 15 sensors in three distinct orientations, measured by the automated platform in Figure 2a.

The automated loading and unloaded process of the single‐finger test platform is illustrated in Figure 2e, and can be watched for 5 successive fingertips in Video S1 (Supporting Information). All 15 fingertips are introduced in **Figure** **3**a (with fabrication details given in Section Details of the 15 Fingertips, and sensing mechanisms in Figures S2– S6, Supporting Information), where they are categorized into three areas:

- **DC Sensors**: which communicate with an Arduino to return up to three information channels from their sensors.
- **AC Sensors**: which use high frequency electrical impedance tomography (EIT) measurements to return up to 1680 information channels in each data frame. The capacitive AC sensor Q1 returns only one information channel, as described in Section Details of the 15 Fingertips.
- **Passive Fingertips**: which are designed to provide task‐specific mechanical properties, but which do not contain any sensing mechanisms.

The five AC sensors A1‐2, F1‐2 and G1 consist of homogeneous hollow shells which are excited by eight electrodes clamped around their neck (Figure 2c; Figure S6, Supporting Information). By collecting thousands of high‐redundancy EIT measurements from the eight electrodes, conductivity changes of the 3D shells can be monitored.^[^ ^46^ ^]^ These conductivity changes can be caused by different mechanisms for different materials, leading to their variabilities along the multimodal sensing axis. For example, fingertips A1 and A2's mechanisms are based on the mobility of the ionic species of the choline chloride that render their organogel piezoresistive. When a mechanical strain is applied to the organogel, the distance between neighboring charges increases, resulting in a decrease in the electrical conductivity. Since only a single cast material is used, their simple design limits delamination risk, reduces cost and fabrication complexity, and provides a pathway to sustainable design. In particular, we draw attention to the biodegradability of the gelatin‐based hydrogel G1,^[^ ^47^ ^]^ and of its ability to be melted down and recast: not only can worn‐down sensors be automatically replaced to eliminate down‐time, but the worn fingertips can further be recycled and reused, or sustainably biodegraded within the lifecycle timescale of Figure 1. AC sensor R1 is also a hollow shell structure, but is a composite of two materials such that its outer surface remains insulated: see Section Details of the 15 Fingertips.

With the 15 fingertips loaded into the rack, the actuated finger can load each finger and automatically initialize the ⩽ 30‐min benchmarking process: the end of Video S1 (Supporting Information) shows this data collection for 3 successive fingertips, during which datasets are built and stored for mechanical properties, force sensitivities, and temperature responsivities. In addition, human responsivities (a fingertip's response to light human contact caused by electrical coupling of the two systems) can be monitored in less than 2 min by applying a 0.2 N normal force onto the pad of a human fingertip (Figure S9, Supporting Information), and localizations can be tested through random probing. In this work, we present and discuss the benchmarking of each of the 15 fingertip's characterization axes. Additionally, we demonstrate how their results can be used to optimize fingertips to new tasks and environments using Figure 1c's two‐finger gripper.

## 3 Results and Discussion

### 3.1 Mechanical Properties

Figure 3b highlights the variability in mechanical responses between all 15 fingertips tested on the automated platform, due to forces from three directions: normal, inward, and outward. All are recorded using Figure 2a's load cell and actuated configuration. This automated process of data collection can be seen in Video S1 (Supporting Information). The soft↔rigid spectrum is clear when a normal force is applied (Figure 3b‐i): those with the steepest force‐displacement gradient (particularly F1, F2, and B1) undergo very little deformation and are therefore capable of transmitting high forces, while those with shallow responses (A2, P2) are better suited to delicate manipulation tasks. To produce sensible results across this soft↔rigid spectrum, the finger's direction of motion is reversed when the first of two conditions is met: a force threshold or a displacement threshold (See Section Automated Benchmarking). Of those falling midway into this spectrum, T2's anisotropic nature is particularly clear from its change in gradient once its uppermost compliant portion has been fully compressed. This resembles the mechanical structure of a human fingertip, where the compliant pad easily deforms before the rigid bone resists further deformations. This gradient shift of T2 can also be seen during the inward loading cycle, where a similar spectrum emerges. The softest fingertips (A1, A2 and P2) still form one distinct end of the spectrum, while the more rigid sensors converge toward a more compliant response than before, with visible hysteresis. This reflects the mechanical stiffness of the fingertip itself in the bent configuration, which remains unchanged as the modular fingertips are changed, and forms the upper stiffness limit of the compliant gripper in this configuration. This effect is even more apparent in the outward configuration, where the responses of all but the softest fingertips are governed by the finger stiffness. Though this provides a morphologically inbuilt compliance to all interactions, this unchanging effect is not the focus of our work and will not be considered in detail. We instead focus on the variable properties of the fingertips themselves, and how these can be optimized to particular tasks. In Figure 1a, passive properties are represented by a soft↔rigid axis, though we note the multidimensional properties that can be selected based on Figure 3's findings: anisotropies and hysteresis.

### 3.2 Force Responsivity

**Figure 4:**
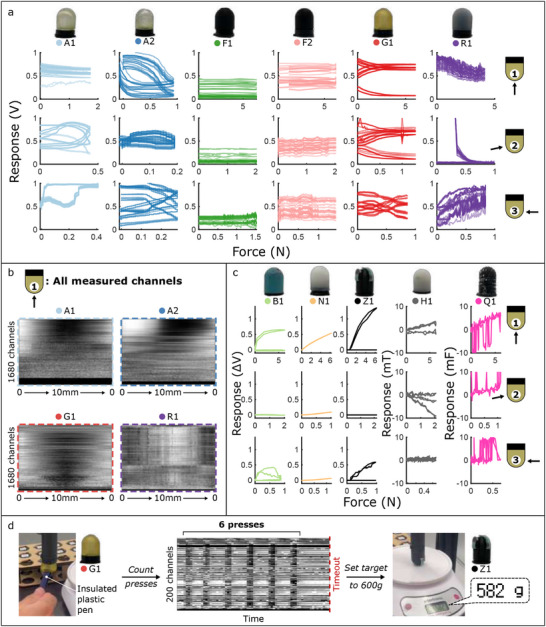
Characterizing force responsivity. a) Most responsive channels of the 6 multiplexed AC sensors during applied normal forces in the three poses. b) Complete responses of selected multiplexed AC sensors in a single load‐unload cycle in pose 1. c) Complete responses of the DC + capacitive sensors during applied normal forces in the three poses. d) Combining sensor‐types in a 2 finger gripper (Video S2, Supporting Information): A deformable AC sensor is used as an interactive input, while a rigid DC sensor applies a target force based on this input.

Beyond their mechanical and morphological properties, 11 of Figure 3a's fingertips are sensorized to achieve tactile interactions, with characteristics varying along three key axes: unimodal↔multimodal responses, sensitivity↔robustness, and location independency↔dependency. The responses of three contrasting sensors (H1, G1 and N1) are shown in Video S1 (Supporting Information), where their most obvious difference is in the number of sensor channels returned. At one extreme, N1's pneumatic chamber monitors just one stimulus, which is highly responsive but does not generate enough information from its single channel to differentiate between different locations or stimuli. H1's 3 channels allow for some directional information to be extracted.^[^ ^48^ ^]^ Conversely, G1's multiplexed measurements monitor 1680 electrode configurations in each frame: though these are highly redundant and coupled, they contain rich information about different stimuli and locations.^[^ ^46^ ^]^ To compare the force characteristics of the six multiplexed AC sensors, **Figure** **4**a plots their top 20 most responsive channels during Figure 3b's normal, inward, and outward tests. Due to their varying materials, compositions, and morphologies, all six behave differently, and can be selected based on the particular task at hand. G1 responds cleanly and near‐linearly to normal forces below 1 N and lateral forces below 0.4 N, before its response plateaus. R1 responds to 5 N of normal force and 1 N of outward force, but suffers from a loose connection when inward forces are applied. Physical effects such as these can be straightforwardly identified through the system's automated physical testing; as soon as continuous monitoring identifies the damage or degradation of a particular fingertip, a functional replacement can be picked up from the rack and installed without the need for human intervention.

The relatively stiff F1 and F2 show little response to the applied force range, while the much more compliant A1 and A2 yield large responses (though the axes have different limits; a standardized‐axes version of these results is presented in Figure S8, Supporting Information). Unlike the others, A1 and A2 show markedly different loading and unloading curves during the testing, which was experimentally observed to be due to their adhesive properties causing them to stick to the surfaces against which they were pressed. Though not explicitly considered during our tests, this kind of surface adhesion can be beneficial in grasping small objects,^[^ ^49^ ^]^ and could form the basis of an additional axis in Figure 1's multiobjective fingertip selection.

Within Figure 4a's top 20‐channel plot, we observe how the multiplexed AC measurements return location‐dependent information from the fingertips. Figure 4b visualizes the raw responses of all 1680 simultaneously monitored channels from four of the fingertips during 10 mm of applied normal deformation. From the triangular pattern of the plot, we see that G1's plateau points actually come at different deformations for different channels, providing an embodied approach for monitoring large deformations of the fingertip without explicitly incorporating sensorized fibers. Fewer channels show visible responses in the remaining three responses: these could instead respond to other stimuli (such as temperature or human touch), or deformations in different locations. Figure 6 will later demonstrate how fingertips G1 and A2 can use these thousands of channels to infer the location of their applied stimuli.

**Figure 5:**
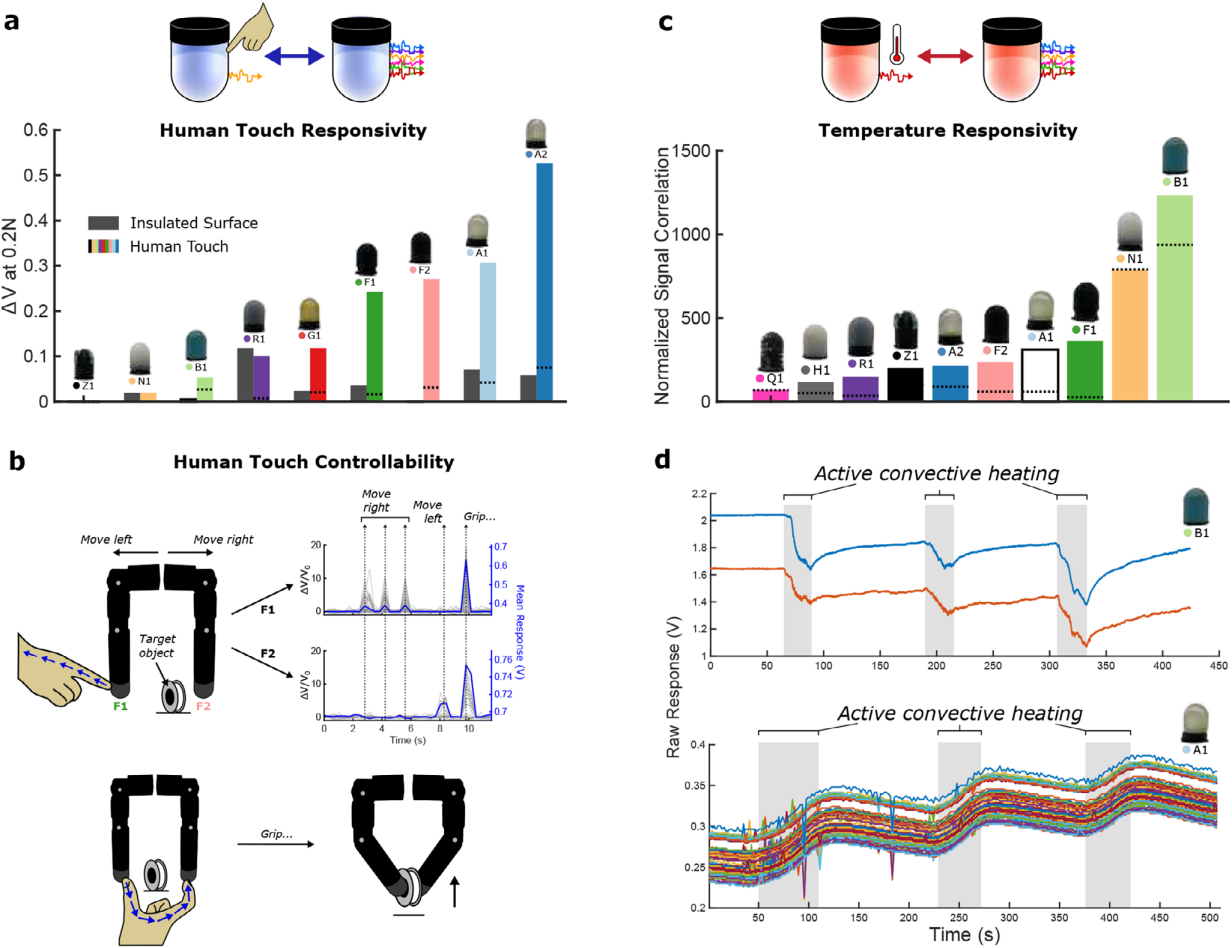
Characterizing temperature and human touch responsivities. a) Raw voltage response magnitudes to a 0.2 N applied force from a human finger vs an insulated surface. Bar height corresponds to the most responsive channel, with a dotted line indicating the mean response of all 1680 channels. b) Using fingertips F1 and F2 to enable interactive human control of a two‐finger robotic gripper (Video S2, Supporting Information). c) Correlations of each sensor's normalized signals to applied fluctuations in temperature. Bar height corresponds to the most correlated channel, with a dotted line indicating the mean correlation of all 1680 channels. d) Raw responses of B1 and A1 during contactless conductive heating.

**Figure 6:**
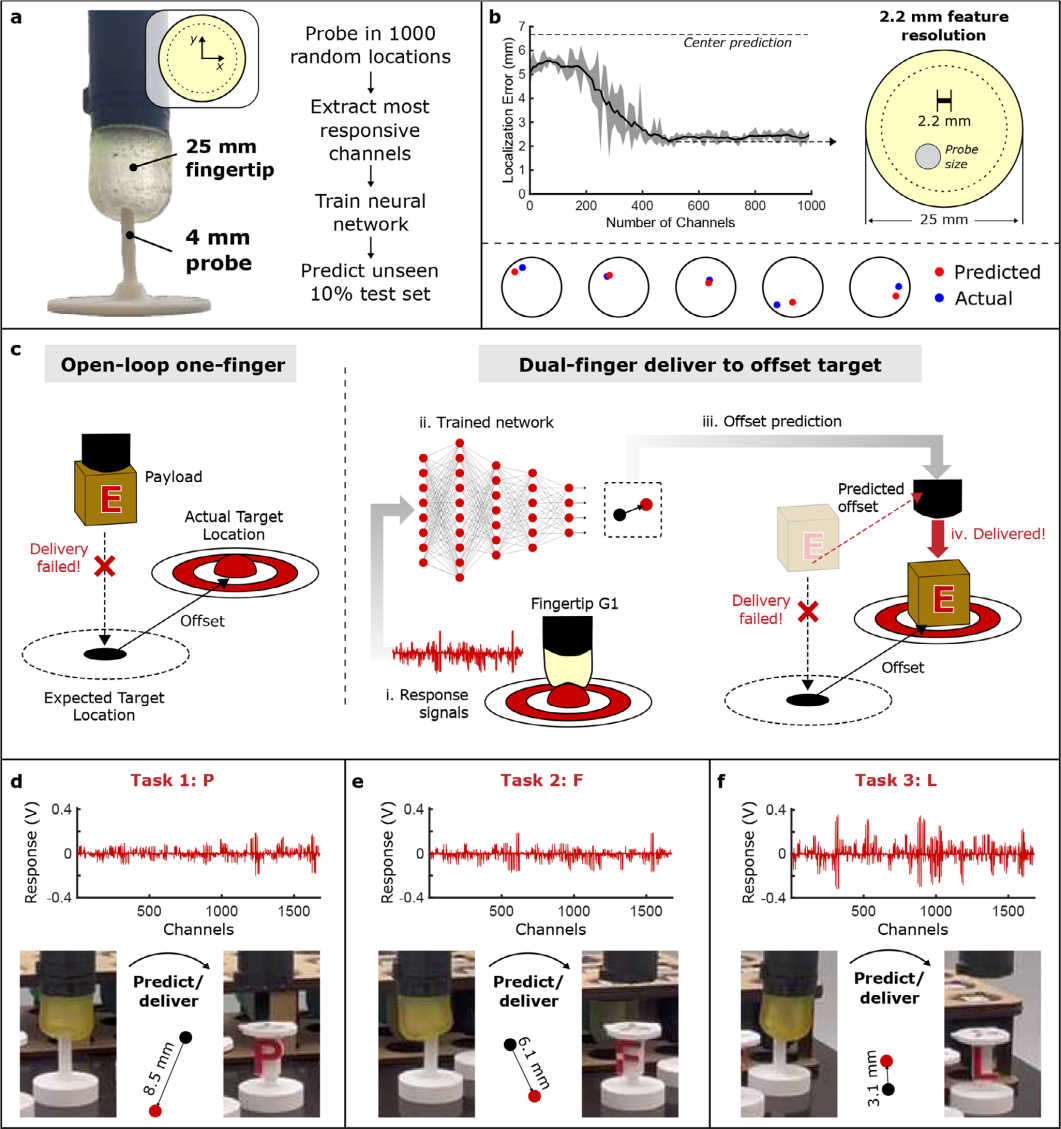
Localization capabilities of different sustainable gels using multiplexed AC measurements. a) Experimental setup: fingertip A2 is pressed in 1000 random locations using a 4 mm probe. b) Resolutions of 2.2 mm can be achieved using fewer than 500 channels. The plot's shaded area represents the standard deviation during convergence. Five consecutive test set predictions are also plotted (see Video S3, Supporting Information). c) Payload delivery to an unknown target position: using one finger to locate the target and another to deliver the payload using the toolchange mechanism. d–f) Target offset predictions for the three letters delivered in Video S3 (Supporting Information), using a network trained to localize probes on fingertip G1.

While these multiplexed measurements contain large amounts of information, they are difficult to extract quantitative predictions from. Figure 4c plots the complete 1, 2, or 3‐channels responses of the remaining five fingertips under the same loading conditions. As seen in Video S1 (Supporting Information), N1 responds cleanly and near‐linearly to applied forces, but without distinguishing their direction. B1 and Z1 respond only at specific transducer locations, and thus are unaffected by applied inward forces. H1 similarly responds only to deformations which physically move its internal magnets, and is thus unaffected by applied outward forces. Though Q1 appears relatively linear in all three directions, its loose connections and subsequently noisy response mean that it is not considered in the rest of this work.

Over the responses visualized in Figure 4a–c, we have seen how different sensing modalities can lead to completely different relative strengths of each of Figure 3a's fingertips, such that different designs are optimal for different tasks and environments. A contrast between the relative strengths of G1 and Z1 is demonstrated in Video S2 (Supporting Information) and Figure 4d's dual‐finger interactive task: a soft, compliant interface (G1) is used for a human to input the desired force which should be applied by the second quantitative fingertip (Z1). Video S2 (Supporting Information) shows the complete process of three inputs and applied forces, to within ±0.3 N accuracy after minimal calibration. This shows the benefits and potential of different fingertip selection within force‐sensing applications: the following section will explore how Figure 3a's fingertips can additionally be employed to detect temperature, human touch, and location‐dependent deformations.

### 3.3 Multimodal Sensorization

Given the 10 kHz AC currents passing through the surfaces of A1‐2, F1‐2, and G1 during sensorization, all five are expected to strongly respond to light human contact.^[^ ^50^ ^]^ Indeed, when the difference between a 0.2 N insulated touch and human touch are compared (**Figure** **5**a), these five sensors dominate by response magnitude. Unsurprisingly, the DC sensors show little or no difference to human touch, as does AC sensor R1, which has an insulating silicone layer between its conductive surface and the human touch. The conductive TPU printed fingertips (F1 and F2) give strong responses to human touch and weak responses to small insulated forces, making them a sensible choice for interactive tasks without the need for stimuli decoupling. One such example using F1 and F2 is shown in Video S2 (Supporting Information) and Figure 5b, in which the position of a dual‐finger gripper can be fine‐tuned by a human operator through light touches in the desired direction of motion. To indicate a “grip” command, both fingertips are touched simultaneously: since this provides a viable path for AC currents to flow between the two, a greater response than touching either fingertip individually is produced (Figure 5b), and the command can be recognized through a simple thresholding check.

In addition, many of the sensorized fingertips are expected to vary their properties with temperature, leading to a viable mechanism for self‐preservation and the detection of external heat sources. The automated testing process for this sensitivity can be seen in Video S1 (Supporting Information): the fingertip is pressed onto a heating element, the readings are left to stabilize, and the temperature is then oscillated between 24 and 38 °C three times over a 20‐min period. Figure 5c plots the correlation between the signal responses and the ground truth temperature measurements. B1 gives a significantly high response. Given its rigidity, Figure 5d checks that this is not due to expansion of the heating element by instead heating the fingertip with a soldering iron which is not in contact with the fingertip. The strong raw response of both channels suggests that its responsivity is governed by material properties of the velostat fabric which it contains, making B1 a good choice for force‐sensitive tasks in which damage‐preventative temperature reflexes are also required. Additionally, AC sensor A1 responds visibly over hundreds of channels; Video S1 (Supporting Information) and Figure 5d visualize the responses of its top 100 ranked channels (see Section 5). These characterizations in Figure 5 begin to convey the wide range of multimodal stimuli which can be selected through the self‐configurable fingertips which we present: given a new stimulus (such as human touch or temperature), characterized fingertips can be selected from their bank which are unresponsive to the stimulus, partially responsive, or largely governed by its effects, depending on the requirements of the task under consideration.

The responses in Figure 4 suggested that the multiplexed AC sensors are capable of returning position‐dependent signals. To explore the extent to which this enables localization, fingertip A2 is pressed to 5 mm depth against an insulated probe of 4 mm diameter in 1000 random *x* − *y* locations: **Figure** **6**a. After ranking the most useful channels (see Section 5), a neural network is trained to predict the locations of a 10% unseen test set, achieving an average error of just 2.2 mm when ≈500 channels are used: Figure 6b. The locations of five consecutive test set predictions are also plotted: Video S3 (Supporting Information) shows the measurement of these five probes among ten consecutive test set predictions.

With such small localization errors from the single‐material homogeneous fingertip, the localization sensitivities of one fingertip can be used to correct error offsets in the dual‐finger gripper. The success of one such task is demonstrated in Video S3 (Supporting Information) and in Figure 6c, which uses fingertip G1 trained in the same way as shown in Figure 6a (see Figure S11, Supporting Information, where average localization errors reach 0.88 mm). Here, four payloads (represented by the capital letters “E,” “P,” “F,” and “L”) must be delivered by one of the fingers to a target, which begins in a known location. For the first payload (“E”), this expected location is not altered, and the fingertip delivers it successfully. However, in subsequent tasks, the target is manually shifted to produce an unknown offset (Video S3, Supporting Information). With an open‐loop delivery system, this would cause these tasks to fail. However, the sensorized fingertip G1 instead probes the expected area to calculate this unknown offset, such that the three payloads can be delivered. The raw magnitudes of the 1680 responses and the three offset predictions of the trained network are shown in Figure 6d–f.

## 4 Conclusion

In this work 15 identically‐shaped fingertips have been fabricated and benchmarked using a novel automated actuated platform. By basing this platform around a self‐configurable robotic gripper, its modularity means that data‐driven sensor selection enables adaptability in task, environment, and lifecycle timescales. **Table** **1** summarizes the automated benchmarking tests which have been performed over Figure 1's 5 axes, including the times taken to characterize each fingertip. In rows 2→4, these are maximum times, corresponding to the displacement limits (and therefore the softest materials) in Section Automated Benchmarking. The softest fingertips can still be benchmarked in a continuous 30‐min period, during which the data is stored in a custom MATLAB class. We have demonstrated how these datasets allow for task‐optimization over fruit picking, guided human/robot interaction, contactless temperature responsivity, localized payload delivery, and force control. During localization benchmarking, sub‐millimeter accuracies of single‐material sensors using EIT have been demonstrated.

**Table 1:** Overview of the automated benchmarking processes which have been presented in this work, for each of Figure 1's task‐optimized selection axes. The full set of results from each fingertip are stored in a custom MATLAB class.

	Characterization Axis	Description	Time Taken
**1**	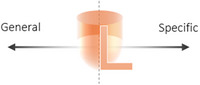	Fully automated fingertip change: mechanically and electrically connecting and disconnecting.	22 s (Video S1, Supporting Information).
**2**	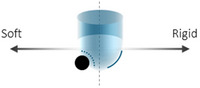	Mechanical characterization of each fingertip in Figure 3: force vs displacement in 3 discrete orientations.	⩽7.5 min (Video S1, Supporting Information with maximum displacements).
**3**	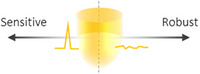	Characterization of force responsivity: signal vs force in 3 discrete orientations.	⩽7.5 min (included in the mechanical tests above).
**4**	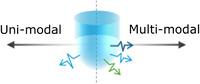	Characterization of human touch responsivity at 0.2 N in Figure 5.	⩽116 s (Video S2 and Figure S9, Supporting Information).
**5**	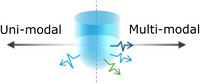	Characterization of temperature responsivity in Figure 5.	20.5 min (Video S1).
**6**	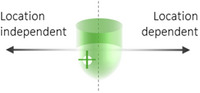	Full collection of 1000‐probe dataset for localization training and testing.	185 min (Gels only, not included in total).
		**TOTAL**	**⩽ 30.3 min**

By developing a self‐configurable sensorized fingertip pipeline, we provide unique benefits to a wide range of tasks, while also promoting a circular economy in robotic manipulation: fingertips can be automatically replaced, repaired or recycled without having to replace the gripper or halt operations. Future implementations could extend to different filtering stages on each finger of a five‐fingered robotic hand, with sensor mechanisms being changed in real time to achieve otherwise impossible tasks. Additionally, future work will aim for the design of fully biodegradable sensorized fingertips when handling biological specimens. The hydrogel fingertips presented here were not yet fully biodegradable due to the PLA/copper base forming their electrode connections: as identified by Hartmann et al.,^[^ ^51^ ^]^ an ultimate goal should be for all constituents to be biodegradable.

Localization was only demonstrated with the sustainable fingertips A2 and G1, and is not included in Table 1's total. We note that this 185 min data collection period trained a neural network from scratch for each new fingertip (see Section Localization Characterization). For more efficient tests of localization, a pretrained network from a similar form factor could instead be used for transfer learning with fewer data points,^[^ ^24^ ^]^ or analytic predictions could be compared.^[^ ^52^ ^]^

Our work has focused on 15 fingertips, covering 8 primary materials and 6 distinct sensing technologies. By including the standard form factor and accompanying code in the supplementary materials, we hope that this platform can be used to characterize and compare a much wider range of technologies, leading to fully self‐configurable and multi‐fingered robotic hands which can selectively filter information to operate across diverse and complex environments.

## 5 Experimental Section

### Details of the 15 Fingertips

The following section briefly describes the fabrication and measurement process for each of the 15 fingertips tested in this work, using the alphanumeric designations from Figure 3a. Each is manufactured to the same dimensions, as shown in Figure S1 (Supporting Information) and the accompanying CAD files.

**DC Sensors**: Pneumatic sensor N1 was cast from Ecoflex 00‐30 silicone, containing a hemispherical chamber of diameter 20 mm positioned 2.5 mm under the skin. The pressure within the chamber was monitored using a MPXH6115AC6U pressure sensor, sealed into the base of the fingertip using Sil‐Poxy glue. Three of the eight electrical connections were used to power and monitor the pressure sensor from the Arduino.

Pressure sensor B1 used piezoresistive material as the core sensing layer. Conductive yarns were arranged on both sides of the resistive layer as electrodes. Specifically, one conductive yarn was placed below the resistor layer as the ground wire. Other conductive yarns (not touching each other) were placed above the resistor layer to form a cross with the ground wire below, and each cross can be considered a touch point. Two touch points were designed into sensor B1; one at the fingertip and the other at 90° offset. The entire outside of the fingertip was wrapped with a nitrile rubber layer for insulation and waterproofing. The resistance between the two connections was monitored by Arduino using a voltage divider with resistor values of 1.5 kΩ.

Pressure sensor Z1 used two circular force‐sensitive resistors, joined to the outside of a rigid (3D printed polylactic acid) shell through a 3 mm layer of Ecoflex 00‐30. The first was positioned to measure forces normal to the tip of the fingertip, and the second at a 45° offset. As with B1, potential dividers were used for monitoring, with resistor values of 4.6 kΩ.

Hall effect sensor H1 contained a 3‐axis hall effect chip (TMAG5273) at its base. Around this, the fingertip was cast from Ecoflex 00‐30 silicone into a 3D printed mold, embedding three 5× 2.5 ×2 mm neodymium magnets. Four of the fingertip's electrical connections were used to power the chip and to collect data through a I^2^C interface with the Arduino.

**Passive Fingertips**: The four passive fingertips of Figure 3a contain no electronics, and were designed to behave with differing mechanical properties.

P1 and P2 were cast into 3D printed molds from Ecoflex 00‐30. P1 was solid silicone, whereas an insert in P2's mold ensured that the fingertip was completely hollow, with a shell thickness of 2 mm.

T1 and T2 were 3D printed using a flexible thermoplastic urethane (Purefil Filament TPU 95A). Their anisotropic structures were generated using the methods proposed by Tricard et al. in ref. [53]: with *z* representing the normal distance from the fingertip's base in their IceSL software, parameters were set to:

(1) $$\begin{array}{cc} \theta =(0,0,0),\;\;\;Infill & =15\%,\;\;\;\phi =\left\{\begin{array}{c} \left(0,0,0\right)\\ \left(0.25,0.25,0.25\right) \end{array}\right.\;\;\;\\ Isotropy & =\left\{\begin{array}{c} (1,1,1),\;\;\;\;\;\;\;z<15.2mm\\ (0,0,0)\;\;\;\;\;\;\;\;\;z\geq 15.2mm \end{array}\right. \end{array}$$

for T1 and:

(2) $$\begin{array}{cc} \theta =(0,0,0),\;\;\;Infill & =15\%,\;\;\;\phi =\left\{\begin{array}{c} \left(0.25,0.25,0.25\right)\\ \left(0,0,0\right)\\ \left(0,0,0\right) \end{array}\right.\;\;\;\\ Isotropy & =\left\{\begin{array}{c} (0,0,0)\;\;\;\;\;\;\;\;\;\;\;\;\;\;\;\;\;\;\;\;\;\;\;\;z<15.2mm\\ (1,1,1)\;\;\;\;\;15.2\leq z<25.4mm\\ (0,0,0)\;\;\;\;\;\;\;\;\;\;\;\;\;\;\;\;\;\;\;\;\;\;\;\;z\geq 25.4mm \end{array}\right. \end{array}$$

for T2, before being fabricated using a Prusa MK3S FDM printer.

**AC Sensors**: With the exception of Q1, all AC sensors worked by generating electrical fields across a hollow shell (wall thickness 2 mm) made from a continuous conductive material, to which eight internal electrodes of copper tape were clamped: Figure 2c. Tetrapolar AC measurements were taken at 10 kHz using^[^ ^54^ ^]^'s serially‐interfacing electrical impedance tomography (EIT) board connected to the eight electrodes. All 1680 possible electrode combinations were monitored per 1.95 s dataframe; though this reduces efficiency by increasing redundancies in the measurements, it enables straightforward data‐driven analysis to find the most responsive electrode configurations, and to account for the effects of noise in particular channels.^[^ ^46^ ^]^

Gelatin hydrogel G1 was selected due to its popularity in soft robotic implementations.^[^ ^46^, ^55^ ^]^ It was cast in a 3D printed mold from a hydrogel of weight composition 1:1.5:1.5:0.2:0.1 gelatin powder (240 bloom, Cake SOS), glycerol (Thermo Fisher Scientific), water, citric acid monohydrate (Thermo Fisher Scientific), and table salt (Sainsbury's). These components were mixed, homogenized at 50°C, cast into the mold, and allowed to solidify for 24 h before removal. A fuller description of the material and its fabrication can be found in ref. [40], where it is shown to remain flexible and stretchable over the timescale of months. Effects of small fingertip fabrication differences on the hydrogel are presented in Figure S10 (Supporting Information).

The organogels A1 and A2 were based on a precursor solution containing 25 wt% acrylamide monomer, N,N‐methylene bisacrylamide (MBA), 1 wt% ammonium persulfate, 7.2 wt% glycerol and 6 wt% choline chloride. Both choline chloride and glycerol form hydrogen bonding with the acrylamide backbone and due to their low volatility, rendering the organogel resistant to drying and freezing when exposed to temperatures as low as ‐20°C, as optimized in [56]. Organogel A1 contained 0.3 wt% MBA and A2 contained 0.6 wt% MBA. The gels were cast in a silicone mould and crosslinked by exposure to 70°C for 1 h.

Shells F1 and F2 were 3D printed from a flexible thermoplastic urethane containing carbon black (Filaflex), with Shore Hardness 92A. F1 used a wall thickness of 2 mm, such that the fingertip effectively behaved as a rigid shell during the experiments performed here. F2 used a wall thickness of 0.5 mm, and could deform during the mechanical characterizations.

R1 was fabricated from two layers: first, a Ecoflex 00‐30 shell with wall thickness 2 mm was cast in a 3D printed mold. Once cured, the shell was turned inside out and its inner surface coated with a flexible conductive thermoplastic elastomer (Shinbon, 30A Shore hardness). Once cured, this was folded back inside the structure, and the layered shell clamped onto the electrodes as in Figure 2c.

Finally, the multilayer capacitive sensor Q1 was constructed with five layers of conformal BCC lattice structures, with conductive fabric layers embedded as positive and grounded electrodes. Conductive yarns were separately connected to each type of electrode. The multilayer structure amplified the capacitive signal generated by the applied contact force. A signal meter collected data at 100 Hz and communicated with the computer at a baud rate of 115 200.

### Automated Test Platform

The modular fingertips were benchmarked using a 3D printed finger with two actuated joints ‐ Figure 2. The 25 mm diameter of each finger allowed them to contain three geared N20 DC motors, controlled through L9110 driver modules and a serially‐connected Arduino Uno.

Two controlled the metacarpophalangeal (MCP) and proximal interphalangeal (PIP) joints using bevel gears and mechanical end stops to move the joints between 0 and 90°. The final motor controlled the automated toolchange mechanism, as described below.

For the single‐finger tests in Figures 3→5, the finger was bolted to a 10 kg load cell via a laser‐cut 5 mm acrylic plate, oriented to record vertical forces through a HX711 amplifier monitored by the Arduino Uno. The load cell was bolted to the gantry of an automated Cartesian frame, using the components of a Creality CR20 pro 3D printer. Using G‐code sent serially from a laptop, this could move the finger in the x/y/z space, and control the temperature of a heated probe (Figure 2a) used for temperature testing.

During the dual‐finger demonstrations of Figures 4d→6, no load cell was used, with both fingers being bolted directly to the printer's gantry, with the two MCP joints being separated by 98.4 mm.

To connect the fingertips, each finger terminated with a magnetic toolchange mechanism. Six neodymium magnets (5× 2.5 ×2 mm) are circularly spaced with alternating polarities (Figure 2d). By rotating the final N20 motor ±60°, these could be aligned to either attract or repel three similarly positioned neodymium magnets of the base of each fingertip. During the attachment and release process, the fingertips were prevented from rotating using three notches in the fingertip rack, which was laser cut from 3 mm plywood to hold 15 fingertips simultaneously.

When a fingertip was attached, eight electrical connections were made between the flat contacts arranged around the fingertip base (Figure 2d) and the spring‐loaded electrical connectors (1.5 mm diameter) contained within each finger. A wire ran from each connector through the finger, and to the breadboard positioned above the setup (Figure 2a). From here, the connections could be accessed by either the Arduino Uno's GPIO pins, or by an EIT board (the open source EIT‐kit by Zhu et al.^[^ ^54^ ^]^) ‐ this depended on the specific fingertip being tested.

### Automated Benchmarking

The data in Figures 3, 4, and 5c can be collected in a completely automated fashion: Video S1 (Supporting Information). After each fingertip was loaded, the fingertip was first normally pressed with a known linear deformation profile against the printer bed while measurements were taken from the load cell and from the fingertip itself. The direction was reversed when either of two criteria was met:

1) The normal force exceeded 6 N.
2) The applied deformation exceeded 10 mm.

The same measurements were then taken for the inward and outward directions by rotating the finger joints to their mechanical limits with the internal N20 motors, then pressing them against normal surfaces (Video S1, Supporting Information). Given the finger's own passive compliance and limits of its magnetic attachment, the limits of these criteria were adjusted to 2 N / 10 mm (inward), and 2 N / 15 mm (outward). Finally, the fingertip was normally pressed onto a 6 mm heating element with ground truth thermistor, controlled and monitored through the printer's serial interface. The fingertips were lowered using 2 N / 15 mm criteria, then the temperature of the probe was oscillated over a 20‐min period (see Figure S7). All recorded data from these automated tests is included in the supplementary material.

In the plots of Figures 4a,b and 6b, the 1680 measured channels were ranked by their response before being plotted. This was achieved using MATLAB's PCA function over each case's time series, then ranking by the absolute magnitude of the 1680 principal components.

The human touch data from Figure 5a was collected by resting the operator's index finger pad‐upward on the printer bed, and normally lowering the fingertip by increments of 0.1 mm until the load cell force registered above 0.2 N, at which point the sensor measurements were taken. In Figure 5a, these measurements are compared to the 0.2 N normal readings on the printer bed from the automated testing process described above. The measurements of all fingertips were recorded in succession under the same environmental conditions. An image of this process can be seen in Figure S9 (Supporting Information).

### Localization Characterization

To create the 1000‐probe dataset of Figure 6a, fingertip A2 was positioned above a 3D printed polylactic acid (PLA) cylindrical probe with 4 mm diameter and a hemisphere‐shaped tip. Point [0, 0, 0] was set to be that at which the probe just made contact with the center of the fingertip. For each probe, *x* − *y* coordinates were independently randomized in the range [‐10, 10] mm, and accepted if the resultant point lay on or within a circle of 20 mm diameter (shown as a dotted line in Figure 6a and b). Given the curvature of the fingertip (R = 12.5 mm) over this area, the z‐motion required for a normal deformation of 5 mm was calculated as:

(3) $$z=R-\sqrt{R^{2}-\left(x^{2}+y^{2}\right)}$$

The responses for each probe were taken to be the 1680 D vector of differences in measured voltage before and during each press, which were ranked using the PCA method described above. A feedforward neural network with tanh activation functions and hidden layer sizes of [100, 50, 20] was used for localization, with an 80:10:10% training:validation:test split. The input layer size matched the number of channels used for localization, and the output layer was of size two: *x* and *y* predictions. This architecture was not optimized, and it is likely that better performances could be reached using the same dataset,^[^ ^52^ ^]^ but provided a consistent method of comparison for different input sizes for the plot in Figure 6b.

In Video S3 (Supporting Information), the E‐P‐F‐L payloads had the same magnetic arrangement as each fingertip, and could each be slotted onto the cuboid target of side length 5.15 mm. Each tube had internal side lengths of 6.5 mm, such that offset errors of up to 1.35 mm could be accommodated without correction: this was less than 50% of any of the calculated offsets in Figures 6d‐f.

## Conflict of Interest

The authors declare no conflict of interest.

## Supporting information

Supporting Information

Supplemental Video S1

Supplemental Video S2

Supplemental Video S3

## Data Availability

The data that support the findings of this study are available in the supplementary material of this article.
