# Supplementary material for: Automated Benchmarking of Variable‐Property Soft Robotic Fingertips to Enable Task‐Optimized Sensor Selection
Source: Adv Sci (Weinh). 2025 Aug 20;13(15):e09991. doi: 10.1002/advs.202509991 (PMC13042928; doi:10.1002/advs.202509991)
Supplement: Supplementary file 1 — Supporting Information [file ADVS-13-e09991-s003.pdf]

Supplementary Material: Automated Benchmarking of Variable-Property Soft Robotic Fingertips to Enable Task-Optimized Sensor Selection

David Hardman Benhui Dai Qinghua Guan
Antonia Georgopoulou Fumiya Iida Josie Hughes

Supplementary Figures

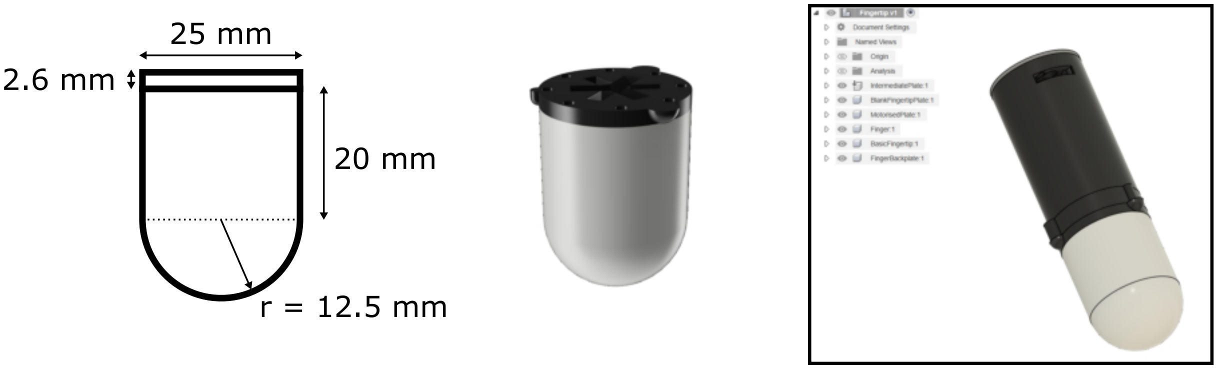

Figure S1: Basic dimensions of the rotationally symmetric fingertips used in benchmarking: these external dimensions are used for all 15 fingertips benchmarked in this work. Internal structures and components differ with each fingertip, as described in the main body of the text. A 2.6 mm thick baseplate forms the mechanical and electrical connections to the finger. A STEP file of the fingertip and lower finger geometries (pictured) is provided in the accompanying repository.

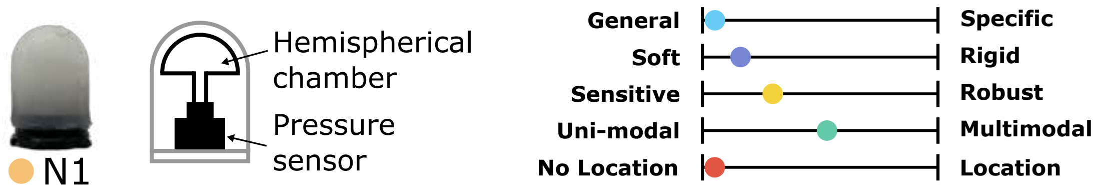

Figure S2: A cross-section of barometric fingertip N1's sensing mechanism and qualitative axis positions. Since the fingertip is soft and sensitive, it is suited to delicate and compliant tasks such as fruit harvesting. However, its inability to detect stimulus location means that it is ill-suited to fine-scale dexterous classification tasks.

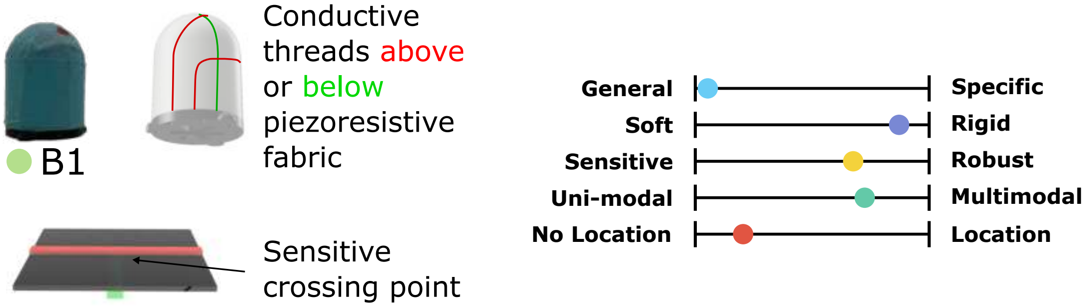

Figure S3: Fingertip B1's 2-point piezoresistive sensing mechanism and qualitative axis positions. The fingertip's rigid nature and temperature dependence means that it is suited to applications requiring precise force and thermal monitoring, such as medical palpation.

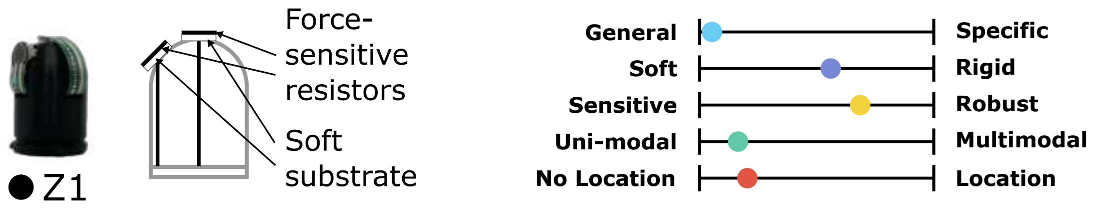

Figure S4: The dual-point sensing mechanism and qualitative axis positions of fingertip Z1, which uses two commercially-available force-sensitive resistors. Its calibrated force-sensitivity and local compliance makes it suitable for precise pick-and-place tasks.

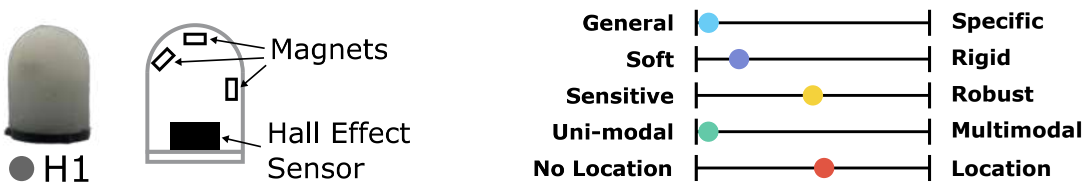

Figure S5: A cross section of magnetic fingertip H1's sensing mechanism and qualitative axis positions. Its 3-axis location-dependence and single (deformation) modality make it suitable for multimaterial shape classifications.

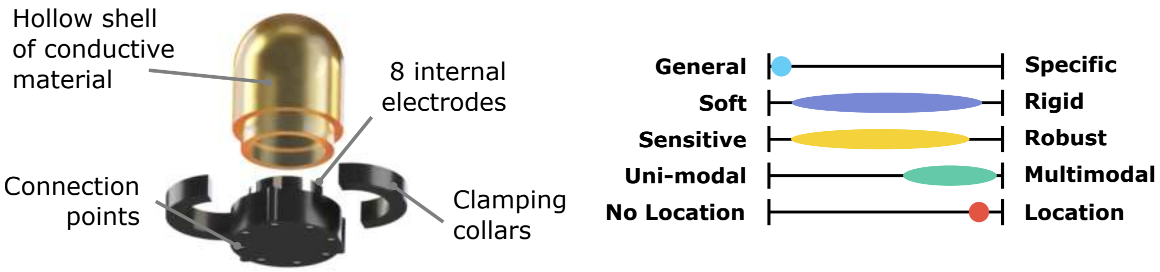

Figure S6: The structure of the EIT fingertips, which monitor thousands of changing impedances across cast conductive shells of a single material. Most axes depend on the material under consideration, but the high signal redundancy tends to lead to multimodal and location-specific signals, making the fingertips suitable for information-rich tasks, such as object localization during changing environmental conditions (such as temperature). When the conductive surfaces are exposed, EIT sensors respond highly in human-contact tasks due to the AC coupling between skin and fingertip.

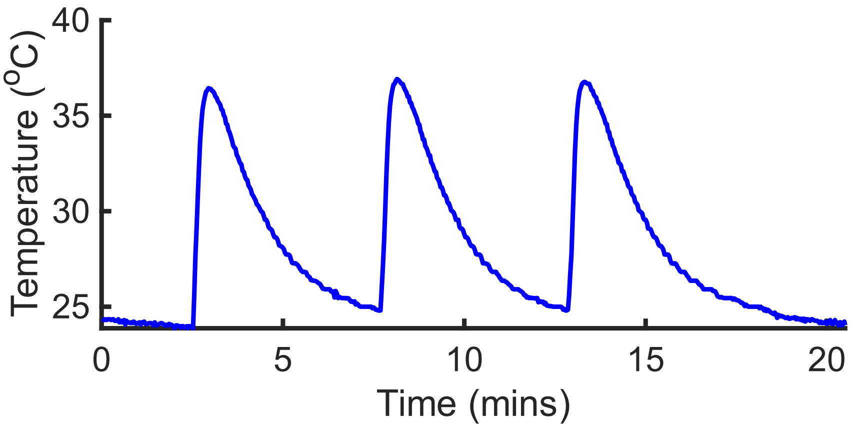

Figure S7: Ground truth temperature probe oscillations during a typical characterization: in this case, the profile from A1's automated benchmarking is used.

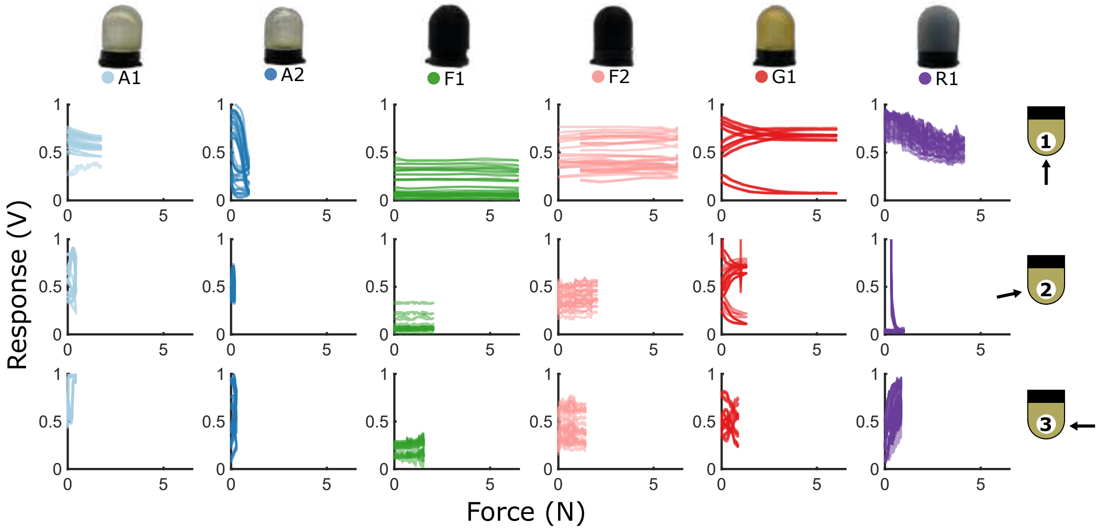

Figure S8: The force responses of Figure 4a presented with standardized x-axis limits: most AC fingertips were sufficiently soft to be reversed by the displacement threshold, through fingertips F1, F2, and G1 met the force thresholds.

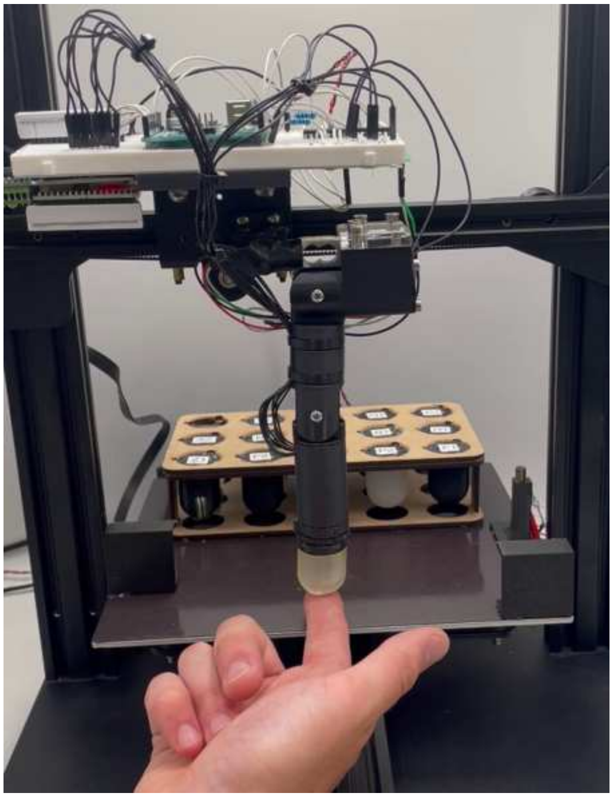

Figure S9: Human touch characterization: each fingertip is lowered onto the pad of the operator's stationary fingertip until the load cell's normal force reaches 0.2 N.

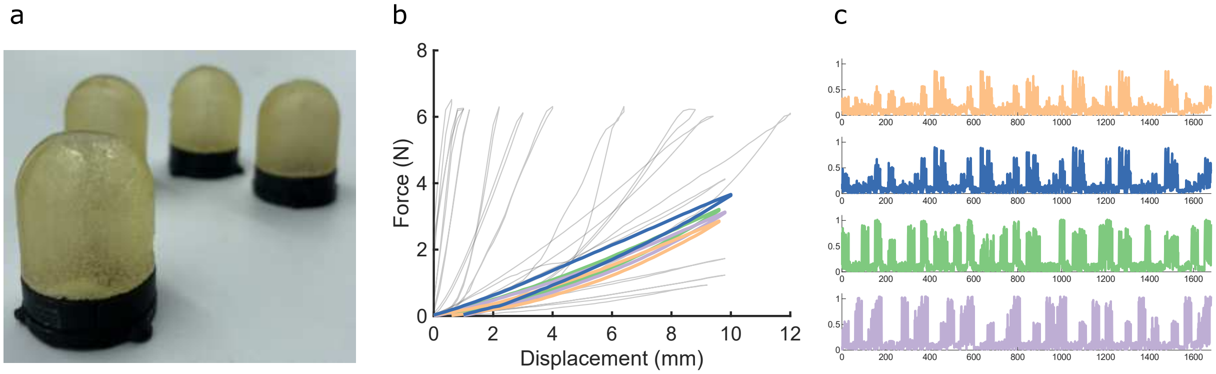

Figure S10: **Hydrogel fabrication effects.** (a) Four hydrogel fingertips are manufactured from the same composition, with any differences in their behaviors arising from fabrication effects and air bubbles. (b) All 4 behave very mechanically similarly during normal testing: the grey lines show the variety of responses from Figure 3b. (c) The raw EIT signals all show similar patterns in their shape, though artefacts of fabrication differences are visible.

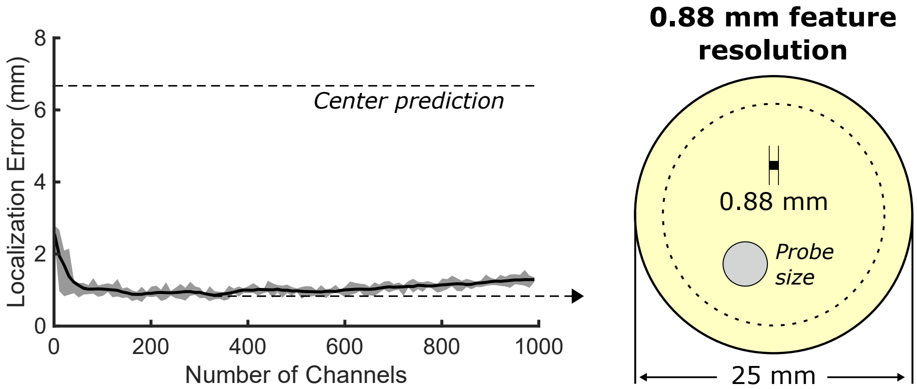

Figure S11: Localization errors of fingertip G1 during training with the process of Figure 6a. The feature resolution reaches a minimum of 0.88 mm.

Supplementary Video Descriptions

- **S1**: An overview of the modular fingertips, showing the automated toolchange/benchmarking processes and how these can be used for task-specific fingertip selection. Three different sensor types (H1, G1 & N1) are compared for similar stimuli.
- **S2**: Two tasks highlighting the selection of fingertips for human interaction: human-controlled gripping, and soft interface devices for force-control inputs.
- **S3**: Localization capabilities of fingertips A2 & G1, corresponding to the data presented in Figure 6.
